# Machine learning for prediction of *Helicobacter pylori* infection based on basic health examination data in adults: a retrospective study

**DOI:** 10.3389/fmed.2025.1587540

**Published:** 2025-06-13

**Authors:** Qiaoli Wang, Tao Liang, Yuexi Li, Peng Zhou, Xiaoqin Liu

**Affiliations:** ^1^Health Management Center, Deyang People’s Hospital, Deyang, Sichuan, China; ^2^Department of Gastroenterology, Deyang People’s Hospital, Deyang, Sichuan, China

**Keywords:** machine learning, *H pylori* infection, basic health examination, SHAP analysis, health examination

## Abstract

**Objective:**

This study aimed to investigate the feasibility of developing machine learning models for non-invasive prediction of *Helicobacter pylori* (*H pylori*) infection using routinely collected adult health screening data, including demographic characteristics and clinical biomarkers, to establish a potential decision-support tool for clinical practice.

**Methods:**

The data was sourced from the adult health examination records within the health management centers of the hospital. The Least Absolute Shrinkage and Selection Operator (LASSO) regression was employed for feature selection. Six distinct machine learning algorithms were utilized to construct the predictive models, and their performance was comprehensively evaluated. Additionally, the SHapley Additive Projection (SHAP) method was adopted to visualize the model features and the prediction results of individual cases.

**Results:**

A total of 10,393 subjects were included in the dataset, with 3,278 (31.54%) having *H pylori* infection. After feature screening, 10 factors were selected for the prediction model. Among six machine—learning models, the Extra Trees model had the best performance, with an AUC of 0.827, Accuracy of 0.744, and Recall of 0.736. The Random Forest model also did well, with an AUC of 0.810. XGBoost attained an AUC of 0.801, indicating moderate predictive capability. SHAP analysis showed that age, WBC, ALB, gender, and wasit were the top five factors affecting *H pylori* infection. Higher age, WBC, wasit and lower ALB were linked to a higher infection probability. These results offer insights into *H pylori* infection risk factors and model performance.

**Conclusion:**

The Extra Trees classifier exhibited the optimal performance in predicting *H pylori* infections among the evaluated models. Additionally, the SHAP analysis enhanced the interpretability of the model, which offers valuable insights for early—stage clinical prediction and intervention strategies.

## Introduction

*H pylori*, a gram—negative bacterium, has the remarkable ability to survive in the harsh acidic environment of the human stomach and colonize the epithelial cells of the gastric mucosa. Globally, *H pylori* is an extremely prevalent pathogen, with nearly half of the world’s population estimated to be infected (1). In China, the prevalence of *H pylori* infection is relatively high. A meta—analysis incorporating 152 studies revealed that the pooled prevalence of *H pylori* in the Chinese mainland was 42.8% (2). *H pylori* infection is a primary cause of various digestive disorders. It is well-established that this bacterium can initiate the development of gastric ulcers, chronic gastritis, duodenal ulcers, and many other digestive system diseases (3). Notably, *H pylori* was classified as a class I carcinogen by the International Agency for Research on Cancer (IARC) (4). People infected with the bacterium *H pylori* have a higher risk of developing stomach cancer, with studies showing that 75% of stomach cancers are caused by *H pylori* infection (5). *H pylori* infection initiates a continuous cascade of progressive alterations within the stomach, ultimately culminating in gastric cancer. The pathological process commences with non-atrophic gastritis (NAG), which then advances to multifocal atrophic gastritis. Subsequently, it progresses to intestinal epithelial hyperplasia, and finally, heteroproliferative changes occur, directly contributing to the development of invasive adenocarcinoma (6–9).

Study shows eradication of *H pylori* infection leads to regression of precancerous lesions (10). Another study reached the conclusion that, in the context of preventing gastric cancer development, eradicating *H pylori* is likely to be most efficacious when the intervention is directed at a younger demographic that has not yet experienced precancerous dysplastic alterations (11). This finding emphasizes the significance of early detection of *H pylori* infection. As such, early—stage diagnosis and timely, appropriate treatment are urgently required. These measures can potentially interrupt the progression from *H pylori* infection to precancerous states and ultimately, gastric cancer, thereby reducing the incidence and burden of this life—threatening disease. Current diagnostic methods for *H pylori* can be classified as invasive or non-invasive. Invasive methods include endoscopy, histology, culture, molecular methods, and the rapid urease test (RUT). Non-invasive methods such as the urea breath test (UBT) and serological tests are also widely used (3). However, some of these methods, regardless of whether they are invasive or non-invasive, are often time—consuming and expensive. There is thus a necessity to develop more effective and accessible screening and diagnostic technologies for *H pylori*. The data obtained from routine health check—ups are easily accessible and play an indispensable role in the early detection of diseases, including *H pylori*—related conditions.

Machine learning (ML) has been extensively applied in medical screening nowadays. A multitude of clinical prediction models leverage machine learning techniques. By analyzing patients’ clinical data, including medical records and biomarkers, these models are capable of predicting disease risks, thereby enabling early—stage and non-invasive screening (12). Liu et al. (13) established several machine learning algorithms for predicting adult *H pylori* infection, all performing well (13). Another study utilized machine learning algorithms to develop a model for predicting Alzheimer’s disease. The results demonstrated that the XGBoost model exhibited the best performance, with an area under the curve (AUC) of 0.915, a sensitivity of 76.2%, and a specificity of 92.9% (14). Moreover, the effectiveness of integrating the XGBoost algorithm in predicting other medical conditions has also been explored. For instance, in a study by Mochurad et al., the utility of the XGBoost algorithm in predicting stroke risk was validated (15). Consequently, healthcare professionals can use machine learning algorithms to better assess risk and take timely preventative action.

Although there are some studies of machine learning algorithms for prediction in *H pylori* (13, 16, 17), there is still a relative lack of large—sample studies based on health examination data in the Chinese adult population. In this study, we aimed to apply ML modeling to predict *H pylori* infection using routine health examination data. We investigated the possibility of estimating *H pylori* infection from basic demographic information, blood test data, etc. with machine learning models. This enables the achievement of the goal that ML—based strategies can be effectively and cost—efficiently applied to improve the detection rate of *H pylori* infection.

## Materials and methods

### Study population and data source

This study adopted a retrospective cross-sectional research design, utilizing data from adults who underwent annual health examinations at the Health Management Center of Deyang People’s Hospital between January 2024 and December 2024. Inclusion criteria: Participants aged 18 years or older who underwent both the C13 urea breath test and routine health examinations at the Health Management Center of Deyang People’s Hospital. Exclusion criteria: ➀ Individuals with a history of digestive system diseases such as gastritis, peptic ulcers, or malignant tumors of the digestive system; ➁ Those with significant cardiac, hepatic, or renal dysfunction; ➂ Patients with other malignant tumors; ➃ Pregnant or lactating women. This study was approved by the Ethics Committee of Deyang People’s Hospital. As this is a retrospective study, patient information was anonymized, thereby waiving the requirement for informed consent.

### Data collection

We extracted relevant data for all eligible participants from the hospital’s health examination database. The database comprises a comprehensive collection of datasets, including demographic information, laboratory test results, and questionnaire responses. Specifically, this study collected detailed data on demographic characteristics (age and gender), lifestyle factors (smoking and alcohol consumption history), clinical measurements (blood pressure), and laboratory parameters (complete blood count, liver function, renal function, and lipid profile), in addition to C13 urea breath test results. In this study, the 13C urea breath test was used to detect *H pylori* infection. The urea breath test (UBT) is the preferred non-invasive detection method, featuring high sensitivity and specificity (18). Before the test, subjects should fast or at least refrain from eating for more than 2 h. Proton pump inhibitors (PPI), H2 —receptor antagonists, and other acid—suppressing agents should be discontinued for 2 weeks, and antibacterial drugs, bismuth—containing drugs, and certain traditional Chinese medicines with antibacterial effects should be discontinued for 4 weeks. A baseline exhaled breath sample is collected by blowing gas into the 0-min air collection bag. After taking the reagent with drinking water, wait for 30 min and then blow gas into the 30-min air collection bag. Connect the 0- and 30-min gas collection bags to a 13C breath analyzer for testing. Whether there is *H pylori* infection is determined by measuring the change in the concentration ratio of ^13^CO_2_/^12^CO_2_ (i.e., the DOB value) in the exhaled breath samples before and after taking the medicine. A DOB value < 4.0 is judged as negative, and a DOB value ≥ 4.0 is judged as positive.

### Data preprocessing and feature selection

In the initial phase, samples with missing values that were missing completely at random (MCAR) were excluded. Subsequently, continuous variables in the dataset were standardized. Meanwhile, binary categorical variables (such as gender, smoking status, and alcohol consumption) were numerically encoded and converted into numerical variables. To enhance the performance of the predictive model, reduce overfitting, and lower computational costs, feature selection was first performed on the dataset to decrease the number of redundant features. In this study, the Lasso regression method was employed for feature selection to identify features strongly associated with *H pylori* infection.

### Management of outcome class imbalance

In the dataset of this study, there were 3,278 cases of *H pylori* infection (accounting for 31.54%) and 7,115 cases of non-infection (accounting for 68.46%). The imbalance in the outcome classes may lead machine learning models to be biased toward predicting the majority class (non-infected cases), thereby achieving high accuracy while neglecting the identification of *H pylori*-positive cases. To address this issue, we employed the Synthetic Minority Over-sampling Technique (SMOTE) to synthesize samples for the minority class, thereby balancing the dataset. This approach aims to provide a more balanced data environment for model training, enhancing the model’s ability to identify *H pylori* infections and improving its overall performance and predictive accuracy.

### Machine learning modeling

The entire study cohort was randomly partitioned into training and validation sets in an 8:2 ratio. In this study, we employed two conventional machine learning algorithms and five ensemble learning algorithms to construct a predictive model for *H pylori* infection. The selected algorithms comprised Logistic Regression (LR), Support Vector Machine (SVM), eXtreme Gradient Boosting (XGBoost), Extra Trees (ET), Random Forest (RF), and Gradient Boosting Tree (GBT). To ensure the optimization of the model performance, we tuned the parameters of each algorithm. Model-specific hyperparameter optimization strategies (Random Search, Bayesian Optimization, and Grid Search) were employed with 5-fold cross-validation to identify optimal parameter configurations for different algorithms, the parameters of the model are reported in Table 1. The performance of the model was comprehensively evaluated by metrics such as accuracy, recall, precision, F1 score, and the Area Under the Receiver Operating Characteristic Curve (AUC). The ROC curve is plotted with the False Positive Rate (FPR) on the horizontal axis and the True Positive Rate (TPR) on the vertical axis, reflecting the performance of the model under different classification thresholds. The AUC is the area under the ROC curve, and its value ranges from 0 to 1. The larger the AUC value, the stronger the model’s ability to distinguish between positive and negative samples, and the better the classification performance. The predictive performance was evaluated through calibration curves and Brier scores, with curves closely aligned to the 45-degree diagonal indicating high concordance between predicted probabilities and actual outcomes, and lower Brier scores reflecting superior calibration quality. Additionally, decision curve analysis (DCA) was applied to quantify the net clinical benefit across decision thresholds. The calculation formulas for each metric are as follows:


$$\mathrm{Accuracy}=\frac{T\,P+T\,N}{T\,P+T\,N+F\,P+F\,N}$$

$$\mathrm{Precision}=\frac{T\,P}{T\,P+F\,P}$$

$$\mathrm{Recall}=\frac{T\,P}{T\,P+F\,N}$$

$$\mathrm{F1}=2\times \frac{P\,r\,e\,c\,i\,s\,o\,n\times R\,e\,c\,a\,l\,l}{P\,r\,e\,c\,i\,s\,i\,o\,n+R\,e\,c\,a\,l\,l}$$

$$\mathrm{FPR}=\frac{F\,P}{F\,P+T\,N}$$

$$\mathrm{TPR}=\frac{T\,P}{T\,P+F\,N}$$

**TABLE 1:** Hyperparameter tuning details for machine learning algorithms.

Models	Hyperparameter search space	Search method	AUC	Selected optimal values
LR	C: [1.e-02 1.e-01 1.e+00 1.e+01 1.e+02	Grid search	0.5982 ± 0.0153	’C’: 1.0, ‘max_iter’: 1000, ‘penalty’: ‘l2’, ‘solver’: ‘liblinear’
SVM	C: [0.1 1 10], kernel: [’rbf’]	Grid search	0.6820 ± 0.0093	’C’: 100.0, ‘gamma’: ‘scale’, ‘kernel’: ‘rbf’
XGB	n_estimators: [100,150], learning_rate:	Bayesian optimization	0.7621 ± 0.0258	’colsample_bytree’: 0.739885182570181, ‘learning_rate’: 0.11064851311347051, ‘max_depth’: 8, ‘min_child_weight’: 1, ‘n_estimators’: 250, ‘reg_alpha’: 0.05866624139366902, ‘reg_lambda’: 0.10294850530885256, ‘subsample’: 0.9987859814917353
ET	n_estimators: [100, 150, 200], max_features: [sqrt, log2], m	Random search	0.8088 ± 0.0047	’n_estimators’: 200, ‘min_samples_split’: 2, ‘min_samples_leaf’: 1, ‘max_features’: ‘sqrt’, ‘bootstrap’: False
RF	n_estimators: [100, 150, 200], max_features: [sqrt, log2], m	Random search	0.7985 ± 0.0044	’n_estimators’: 200, ‘min_samples_split’: 2, ‘min_samples_leaf’: 1, ‘max_features’: ‘log2’, ‘class_weight’: None, ‘bootstrap’: False
GBT	n_estimators: [100, 150, 200], learning_rate: [0.05, 0.1, 0.	Random search	0.7967 ± 0.0070	subsample’: 1.0, ‘n_estimators’: 150, ‘min_samples_split’: 5, ‘min_samples_leaf’: 2, ‘max_features’: ‘log2’, ‘max_depth’: 7, ‘learning_rate’: 0.2

### Result visualization and feature importance assessment

After constructing the prediction model using the best-performing machine learning algorithm, this study employs the SHAP (SHapley Additive exPlanations) method to evaluate the contribution of each feature to the prediction results. SHAP, as a state-of-the-art approach for enhancing the interpretability of tree-based models, utilizes a game-theoretic framework to aggregate the local contributions of individual features, thereby providing a global explanation of the model’s behavior. Compared to other feature importance evaluation methods, SHAP not only quantifies the contribution of each feature to the prediction outcome but also indicates whether the influence of each predictor variable on the target variable is positive or negative. This capability allows us to further determine whether a specific feature acts as a protective factor or a risk factor.

### Statistical analysis

The statistical analysis in this study was performed in the Jupyter environment using Python version 3.12.7. Continuous variables were described as mean ± standard deviation (SD), and between-group comparisons were conducted using unpaired *t*-tests or other appropriate tests. Categorical variables were analyzed using chi-square tests, and stepwise logistic regression was employed for multifactorial analysis. Data standardization was implemented using the StandardScaler function from the Scikit-learn package. A *p* < 0.05 was considered statistically significant.

## Results

### Baseline characteristics

The study cohort comprised 10,393 eligible participants, with a gender distribution of 5,644 males (54.31%) and 4,749 females (45.69%). Table 2 presents comparative demographic and clinical characteristics between *H pylori*-positive (*n* = 3,278) and negative (*n* = 7,115) subgroups within the total cohort (*N* = 10,393). Univariate analysis demonstrated statistically significant intergroup differences (*P* < 0.05) in variables including gender, age, smoking history, drinking history, BMI, waist circumference, SBP, DBP, TP, ALB, UA, CRE, FPG, HDL, LDL, TC, ANC, AMC, ALC, WBC, and Hb.

**TABLE 2:** Univariate analysis of risk factors associated with *H pylori* infection.

Feature	*H pylori* ( +)	*H pylori*(−)	*p*-value
Gender			<0.001
Male	1883	3761	
Female	1395	3354	
Smoking			<0.001
Yes	749	1345	
No	2529	5770	
Drinking			<0.001
Yes	664	1149	
No	2614	5966	
Age (years)	45.656 @ 12.034	42.885 @ 12.503	<0.001
BMI (kg/m^2^)	23.933 @ 3.407	23.525 @ 3.303	<0.001
Waist (cm)	82.520 @ 10.741	80.957 @ 10.540	<0.001
SBP (mmHg)	122.533 @ 16.345	121.158 @ 15.585	<0.001
DBP (mmHg)	73.883 @ 11.256	72.888 @ 10.903	<0.001
TP	74.429 @ 3.833	74.871 @ 3.811	<0.001
ALB	46.457 @ 2.540	46.923 @ 2.555	<0.001
ALT	25.573 @ 20.525	25.137 @ 23.664	0.363
AST	24.211 @ 11.488	23.911 @ 10.936	0.201
UA	355.971 @ 94.601	351.748 @ 95.824	0.036
CRE	70.263 @ 15.231	69.203 @ 23.114	0.017
BUN	5.066 @ 1.256	5.067 @ 1.406	0.964
FPG	5.243 @ 1.350	5.136 @ 1.170	<0.001
HDL	1.399 @ 0.360	1.429 @ 0.371	<0.001
LDL	2.767 @ 0.700	2.709 @ 0.706	<0.001
TC	4.857 @ 0.881	4.812 @ 0.916	0.017
TG	1.713 @ 1.563	1.674 @ 1.641	0.254
ANC	3.616 @ 1.155	3.462 @ 1.152	<0.001
AMC	0.380 @ 0.129	0.370 @ 0.129	<0.001
ALC ( × 10^9^/L)	2.050 @ 0.592	1.985 @ 0.572	<0.001
RBC ( × 10^12^/L)	4.887 @ 0.534	4.906 @ 0.550	0.098
WBC ( × 10^9^/L)	6.225 @ 1.522	5.990 @ 1.524	<0.001
Hb	147.370 @ 15.992	146.664 @ 15.771	0.0346
PLT ( × 10^9^/L)	204.593 @ 67.226	205.328 @ 59.293	0.573

BMI body mass index; SBP, waist circumference, systolic blood pressure; DBP, diastolic blood pressure; TP, total protein; ALB, albumin; UA, uric acid; CRE, creatinine; FPG, fasting plasma glucose; HDL, high-density lipoprotein cholesterol; LDL. low-density lipoprotein cholesterol; TC, total cholesterol; TG, Triacylglycerol; ANC, absolute neutrophil count; AMC, absolute monocyte count; ALC, absolute lymphocyte count; RBC, Red blood cell count; WBC, white blood cell count; Hb, hemoglobin; PLT, platelet.

### Stepwise multivariate logistic regression

The stepwise Logistic Regression model showed that gender, drinking, age, ALB, ANC, AMC, and ALC were independent influencing factors of *H pylori* infection (*P* < 0.05). Specifically, male gender, a history of drinking, increasing age, higher ANC, and higher ALC were positively associated with *H pylori* infection. In contrast, female gender and higher levels of ALB and AMC were associated with a reduced risk of *H pylori* infection. Refer to Table 3 for more details.

**TABLE 3:** Stepwise multivariate logistic regression analysis of factors associated with *H pylori* infection.

Feature	Coef	SE	*z*	*p*-value	OR	95%CI
Gender	0.145	0.049	2.966	0.003	1.156	1.050–1.272
Drinking	0.174	0.059	2.941	0.003	1.190	1.060–1.337
Age	0.0154	0.002	8.486	<0.001	1.016	1.012–1.019
ALB	-0.064	0.009	-7.052	<0.001	0.939	0.922–0.955
ANC	0.131	0.022	6.057	<0.001	1.139	1.092–1.189
AMC	-0.596	0.213	-2.792	0.005	0.551	0.363–0.837
ALC	0.224	0.040	5.588	<0.001	1.251	1.157–1.354

### Feature selection

**FIGURE 1:**
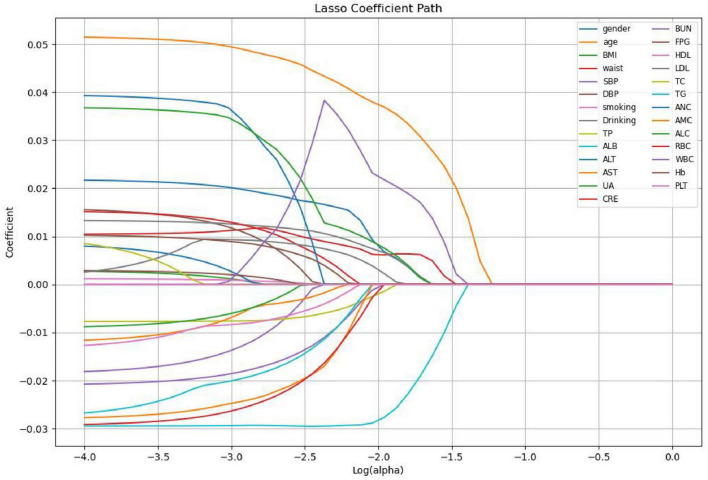
LASSO coefficients of all 27 features.

**FIGURE 2:**
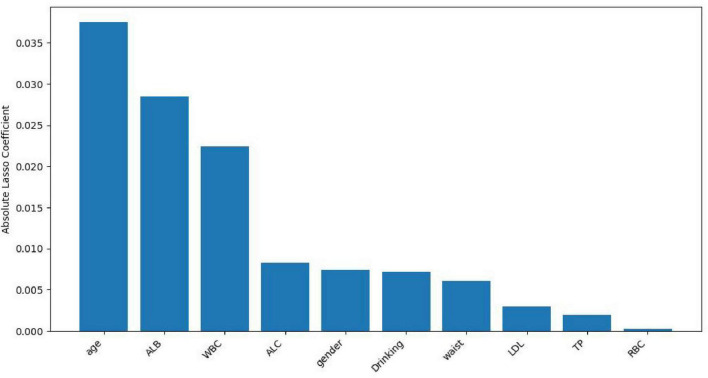
Plots of absolute values of lasso coefficients for the remaining 10 features after feature selection.

Lasso regression screening identified 10 features associated with Hp infection, namely gender, age, waist, Drinking, TP, ALB, LDL, ALC, RBC, WBC. These features play a crucial role in differentiating between *H pylori*- positive and *H pylori*- negative individuals in the population. Figure 1 illustrates the relationship between the coefficients and C (the inverse of the regularization strength) under Lasso L1 regularization. This visualization reveals that numerous variables are non-essential. Omitting these non-essential variables can enhance model performance and prevent overfitting. Figure 2 shows the absolute values of lasso coefficients for the remaining 10 features after feature selection.

### Machine learning model construction

**FIGURE 3:**
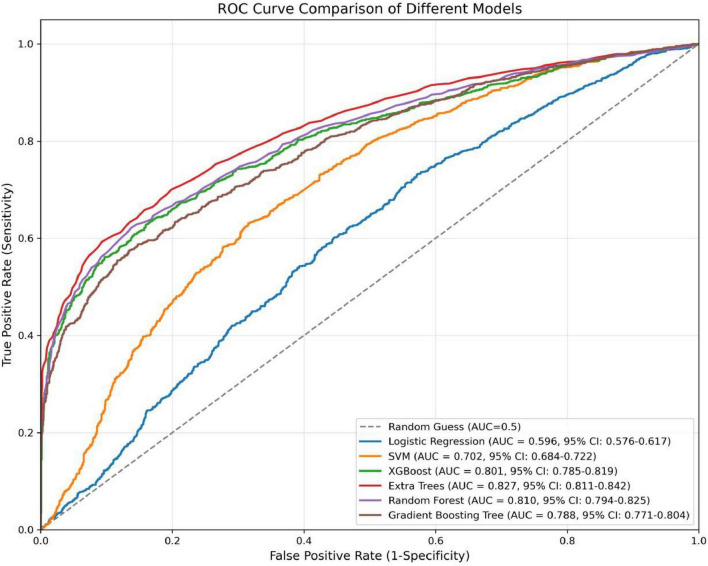
Receiver operating characteristic (ROC) curve of the predictive model.

**FIGURE 4:**
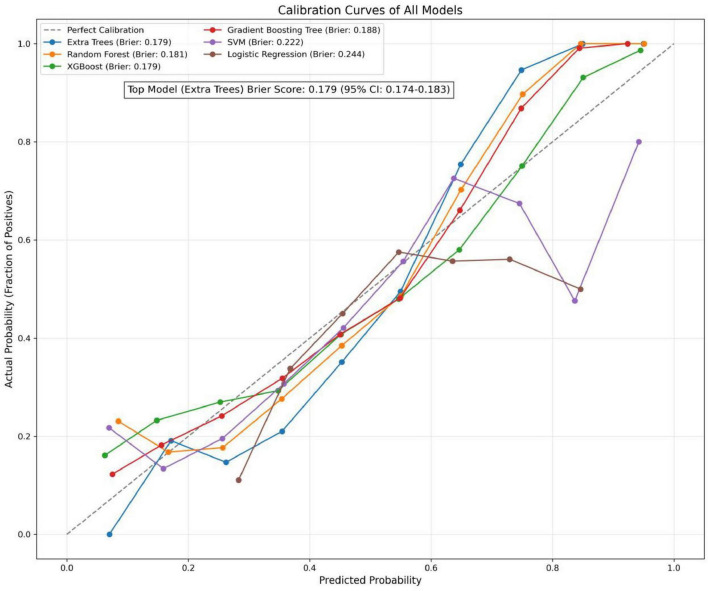
Calibration curves of all models.

**FIGURE 5:**
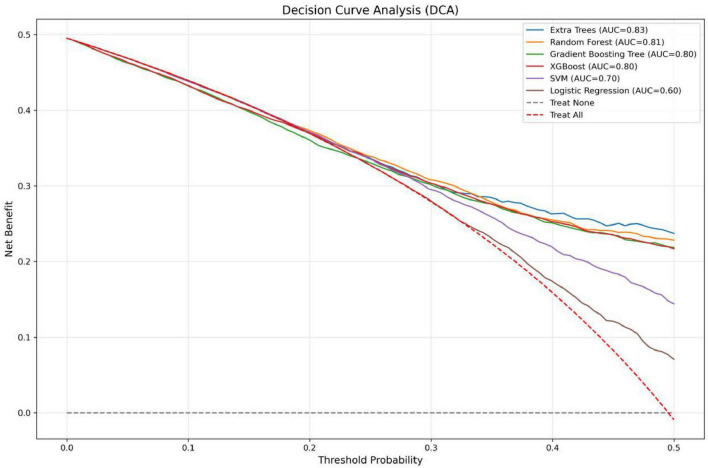
Decision curve analysis (DCA).

In the present study, we developed a total of six machine—learning models using ten pre-selected features. These models consisted of Logistic Regression, Support Vector Machine (SVM), eXtreme Gradient Boosting (XGBoost), Extra Trees, Random Forest, and Gradient Boosting Tree. Subsequently, we evaluated the performance of these models using various metrics in the test set (Table 4). Figure 3 shows the Receiver Operating Characteristic (ROC) curves of each model. Among these seven models, the Extra Trees model outperformed other models in terms of key evaluation metrics, namely accuracy, recall, F1 score, and the AUC. Specifically, it achieved an accuracy of 0.744, a recall of 0.736, an F1 score of 0.740, and an AUC of 0.827. Additionally, it demonstrated high precision, with a value of 0.746. After a comprehensive consideration of all the evaluation parameters, the Extra Trees model was identified as the optimal model for predicting *H pylori* (Hp) infection. This finding indicates that the Extra Trees model exhibits superior performance in the prediction of Hp infection compared to the other models evaluated in this study. The Random Forest model demonstrated robust discriminative performance, achieving an area under the receiver operating characteristic curve (AUC) of 0.810. Additionally, it exhibited strong classification metrics: accuracy = 0.728, recall (sensitivity) = 0.494, precision = 0.741, and F1-score = 0.717. Among the evaluated models, XGBoost achieved an AUC of 0.801. The SVM model showed a moderate performance. In contrast, the LR model underperformed across all evaluation metrics. It only achieved an AUC of 0.596, an accuracy of 0.575, a recall of 0.577, a precision of 0.570, and an F1 score of 0.574. In addition, the calibration curve (Figure 4) indicated that Extra Trees provided the best calibration performance at 0.179 (95% CI: 0.174–0.183). Furthermore, in the Decision Curve Analysis (DCA) of the validation set, all these models demonstrated better net clinical benefits compared to the “Treat None” and “Treat All” strategies. Among them, Extra Trees, with its relatively high AUC and performance on the curve, performed excellently in risk stratification and prediction. These are presented in Figure 5.

**TABLE 4:** Metrics for performance assessment of each predictive model.

Models	AUC	Accuracy	Recall	Precision	F1 Score
LR	0.596(0.576–0.617)	0.575(0.557–0.593)	0.577 (0.552–0.603)	0.570 (0.546–0.597)	0.574 (0.551–0.593)
SVM	0.702 (0.684–0.729)	0.649 (0.633–0.666)	0.717 (0.692–0.741)	0.628 (0.606–0.652)	0.670 (0.650–0.689)
XGB	0.801 (0.785–0.819)	0.726 (0.709–0.742)	0.691 (0.667–0.716)	0.740 (0.716–0.763)	0.709 (0.695–0.734)
ET	0.827 (0.811–0.842)	0.744 (0.728–0.759)	0.736 (0.713–0.758)	0.744 (0.721–0.767)	0.740 (0.722–0.758)
RF	0.810 (0.794–0.825)	0.728 (0.712–0.745)	0.694 (0.670–0.720)	0.741 (0.717–0.762)	0.717 (0.698–0.736)
GBT	0.788 (0.772–0.804)	0.708 (0.692–0.725)	0.671(0.646–0.695)	0.721 (0.696–0.744)	0.695 (0.675–0.715)

### Visualization of results: importance ranking and SHAP values

**FIGURE 6:**
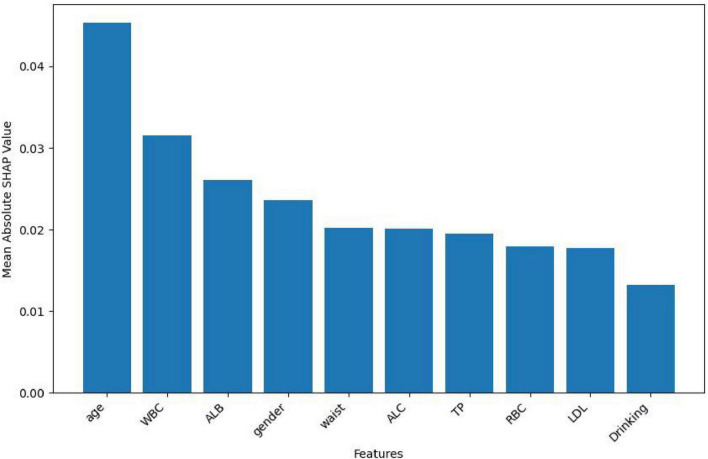
Extra trees model SHAP feature importance.

**FIGURE 7:**
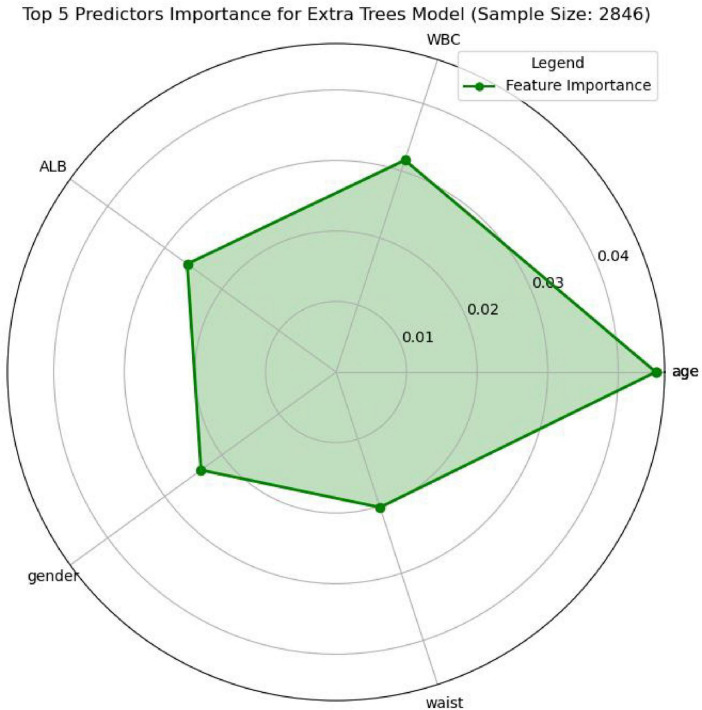
Radar plot for the top 5 predictors importance of *H pylori* infection.

In this study, the SHapley Additive exPlanations (SHAP) method was employed to evaluate the importance of features. To gain a deeper understanding of how individual features contribute to the prediction outcomes in the *H pylori* infection prediction model based on the Extra Trees algorithm, we calculated the SHAP value for each feature. First, we analyzed the feature importance in the Extra Trees model. The results showed that the 10 features were ranked in the following order of importance: age, WBC, ALB, gender, waist, ALC, TP, RBC, LDL, drinking (Figure 6). To visually present the relative importance of the features more intuitively, we used radar charts to visualize the top five predictors. As shown in Figure 7, age, WBC, ALB, gender, and wasit were the most influential factors in the model.

**FIGURE 8:**
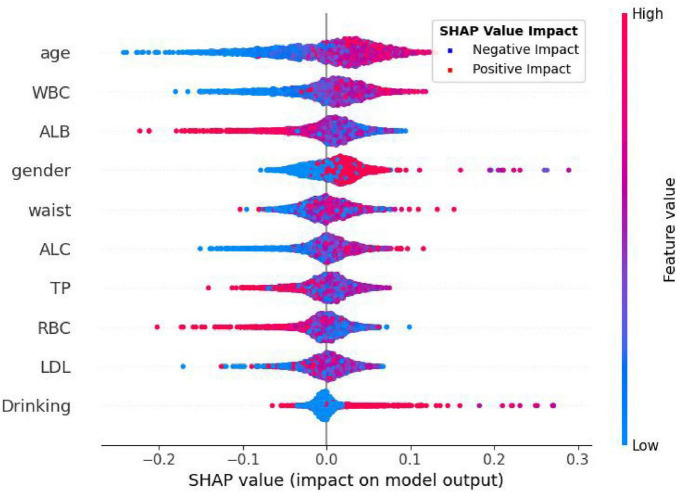
The SHAP summary plot.

In Figure 8, each dot in each row represents the impact of each characteristic on *H pylori*—infected individuals. These characteristics are listed in descending order of importance: age, WBC, ALB, gender, waist, ALC, TP, RBC, LDL, drinking. As shown in the figure, the Extra Trees model ranks age as the most important characteristic. There is a positive association between the feature value and its predictive effect on *H pylori* infection. Specifically, a higher feature value has a more positive impact on predicting *H pylori* infection, whereas a lower value contributes less. For the output of the *H pylori* infection predictive model, patients with higher values of age and WBC are more likely to be predicted by the model as having *H pylori* infection. Conversely, lower ALB values indicate that the model predicts a higher probability of *H pylori* infection.

**FIGURE 9:**
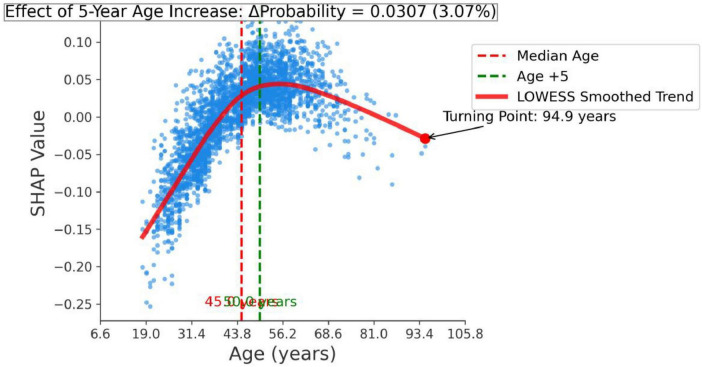
Effect of age on helicobacter pylori infection probability.

In addition, we performed a feature effect analysis on the most important feature, age, as shown in Figure 9. The infection probability of *H. pylori* exhibits an age-dependent increase, with each incremental 5-year age elevation at the median level associated with an average 0.0307-unit rise in predicted probability.

## Discussion

In this study, we identified significant differences between the two groups regarding gender, age, smoking history, alcohol consumption, BMI, waist circumference, systolic blood pressure (SBP), diastolic blood pressure (DBP), total protein (TP), albumin (ALB), uric acid (UA), creatinine (CRE), fasting plasma glucose (FPG), high-density lipoprotein cholesterol (HDL-c), low-density lipoprotein cholesterol (LDL-c), total cholesterol (TC), absolute neutrophil count (ANC), absolute monocyte count (AMC), absolute lymphocyte count (ALC), white blood cell count (WBC), and hemoglobin (Hb). Subsequent traditional stepwise logistic regression analysis revealed that male gender, a history of alcohol consumption, advanced age, elevated ANC, and increased ALC were positively associated with *H pylori* infection. Conversely, female gender, higher albumin levels, and elevated AMC were associated with a reduced risk of *H pylori* infection. These findings further underscore the gender disparity in *H pylori* infection, consistent with previous studies reporting a slightly higher prevalence in men (44.1%) compared to women (41.6%) (2). Previous studies have demonstrated a correlation between *H pylori* infection and various inflammatory mediators. Notably, these studies observed elevated neutrophil levels in *H pylori*-positive populations, as well as an association between increased age and a higher prevalence of *H pylori* infection (19, 20). These research findings strongly support the involvement of neutrophils in the inflammatory process induced by *H pylori* infection. *H pylori* typically colonizes the less acidic gastric antrum, a behavior that recruits neutrophils and lymphocytes to the site of infection. The bacterium’s encoded virulence factors, cytotoxin-associated protein A (CagA) and vacuolating cytotoxin A (VacA), upregulate the expression of pro-inflammatory cytokines by activating the NF-κB signaling pathway. This mechanism triggers a systemic inflammatory response, underscoring the critical role of neutrophils in the pathogenesis of *H pylori*-related inflammation (21–23). In addition, several studies have demonstrated a negative association between albumin levels and *H pylori* infection, indicating that lower albumin levels may serve as an independent risk factor for *H pylori* infection (24–26). Our findings are consistent with these observations. *H pylori* infection may impair gastric digestion and absorption by reducing albumin levels, potentially mediated through an increase in gastric pH. Furthermore, the damage to the gastric mucosa caused by *H pylori* infection can disrupt digestive and absorptive functions, leading to decreased serum albumin levels.

This study is the first to explore the predictive potential of routine physical health examination indicators for detecting *H pylori* infection, offering a valuable tool for early intervention and effective screening. Unlike previous studies on *H pylori* infection prediction, which often relied on single features or simplistic models, this study developed and compared six machine learning models to predict *H pylori* infection using limited health check-up data. Compared with previous models for predicting Helicobacter pylori infection, we incorporated the Extra Trees algorithm (13, 16, 17). Additionally, rigorous data preprocessing and feature selection were employed to effectively mitigate issues of overfitting and feature redundancy, ensuring the robustness and reliability of the models. Among the six machine learning classifiers evaluated, the Extra Trees model demonstrated the best performance. This superiority can be attributed to its high degree of randomness in selecting samples, features, and splitting points, which enhances the diversity among decision trees and improves generalization ability. Additionally, the Extra Trees model exhibits strong robustness to noisy data, effectively mitigating overfitting while maintaining high training efficiency. In addition, the Random Forest, XGBoost, and Gradient Boosting Tree models also demonstrated high AUC values, indicating strong predictive performance. In one study, researchers developed and evaluated several machine learning prediction models, including K-Nearest Neighbors (KNN), Logistic Regression with Lasso Penalty (LR), Support Vector Machines (SVM), Random Forests (RF), Naive Bayes (NB), and XGBoost (XGB). The results indicated that the XGBoost method performed slightly better than the other classifiers. However, RF demonstrated comparable accuracy, with both models achieving AUROC scores ranging from 0.78 to 0.79 (16). However, in our study, we observed that the Extra Trees algorithm outperformed other models, achieving the highest AUROC of 0.827, followed by Random Forest with an AUROC of 0.810. This discrepancy in model performance may be attributed to differences in dataset characteristics, feature engineering, or hyperparameter tuning. Nonetheless, our findings consistently support the notion that ensemble algorithms generally exhibit superior performance in similar tasks. The superior performance of the ensemble models in this study can be attributed to the high complexity and non-linear relationships inherent in the data, which these models are well-equipped to handle. Additionally, ensemble methods can automatically filter features and evaluate their importance during the model-building process. This capability is critical for identifying key factors from large datasets and enables more efficient utilization of data. Consistent with our findings, another study also demonstrated that Random Forest (RF) models are the optimal choice for detecting *H pylori* infection using routine blood tests, achieving an ROC of 0.951 (17), the performance of our model was slightly lower than that of the aforementioned study, our approach utilized a broader range of routine health examination data for screening, including indicators related to liver function, blood lipids, and kidney function. Differences in data types, model complexity, and demographic characteristics may account for the variations in performance metrics across studies. Nevertheless, our findings demonstrate that these machine learning predictive models can serve as effective tools for predicting *H pylori* infection during routine health check-ups. In contrast, the Logistic Regression (LR) model performed poorly, indicating its limited ability to distinguish between *H pylori*-positive and -negative cases, likely due to its inability to capture the complexity of the data. This is consistent with a previous study, which also reported suboptimal performance of an LR model constructed to predict *H pylori* infection using routine blood test indicators, achieving an ROC of 0.596 and an accuracy of 0.575 (17). In addition, we further evaluated the model’s clinical utility through decision curve analysis (DCA). The Extra Trees model demonstrated robust performance in risk stratification and prediction, characterized by relatively high AUC values and favorable decision curve outcomes. These findings not only corroborate its discriminative capacity but also underscore its potential to guide individualized treatment strategies, thereby supporting its clinical applicability.

SHapley Additive exPlanations, as a method for interpreting ML model predictions, provides valuable insights into the prediction process and the contribution of individual features. In this study, the Extra Trees classifier demonstrated the best performance; therefore, we used the Extra Trees algorithm to rank features by importance and visualized the results using radar plots and SHAP summary plots. The analysis revealed that the top five most important features were age, WBC, ALB, gender, and wasit, all of which showed strong associations with *H pylori* infection. Notably, the prevalence of *H pylori* infection increased gradually with age, which is consistent with previous studies (2, 27, 28). The gradual increase in *H pylori* infection with age may be attributed to age-related declines in immunity, which elevate the risk of infection. Additionally, *H pylori* infection prevalence varies by gender, with slightly higher rates observed in men compared to women (2). Changes in blood test markers may reflect the body’s immune status and inflammatory response, which are associated with *H pylori* infection. Although *H pylori* itself cannot enter the bloodstream, the bacteria can trigger the release of inflammatory mediators into the circulation, leading to a systemic inflammatory response. This process may contribute to the development of various systemic diseases (29). Albumin levels can serve as an indirect indicator of nutritional status. Previous studies have demonstrated a significant correlation between lower albumin levels in *H pylori*-positive individuals compared to those who are *H pylori*-negative (30, 31). These findings suggest that *H pylori* infection may contribute to reduced serum albumin levels. Our findings are consistent with previous studies and further validate the significant role of these factors in *H pylori* infection.

This study has some limitations. As a single-center retrospective study in Sichuan Province, the findings acknowledge that they may have selection bias and limited generalizability, potentially failing to be extrapolated to other regions or ethnic groups. Furthermore, potentially influential covariates of Helicobacter pylori infection—including educational attainment, dietary patterns, lifestyle factors (e.g., tea and soft drinks), and medication histories (notably PPIs/antibiotics) (32–34)—were not systematically captured in our dataset. This critical gap may substantially constrain the model’s clinical utility by underestimating multifactorial infection dynamics. This omission might compromise the model’s comprehensiveness. Future studies should expand sample sizes, adopt multicenter designs, and include more clinical and lifestyle data to improve accuracy. Enhancing model interpretability also remains a key challenge for supporting clinical decision-making.

## Conclusion

In this study, we developed a machine learning model to predict *H pylori* infection using routine health examination data. The Extra Trees classifier performed best, while other ensemble models (Random Forest, XGBoost and Gradient Boosting Tree) also outperformed traditional logistic regression. These models excel at handling complex data relationships and provide a valuable tool for early clinical prediction of *H pylori* infection. Using SHAP analysis, we identified the top five most important factors: age, WBC, ALB, gender, and wasit. These findings offer insights for further research into *H pylori* infection mechanisms and prevention strategies.

## Data Availability

The raw data supporting the conclusions of this article will be made available by the authors, without undue reservation.

## References

[1] Chen Y Malfertheiner P Yu H Kuo C Chang Y Meng F Global prevalence of Helicobacter pylori infection and incidence of gastric cancer between 1980 and 2022. *Gastroenterology.* (2024) 166:605–19. 10.1053/j.gastro.2023.12.0222 38176660

[2] Xie L Liu G Liu Y Li P Hu X He X Prevalence of *Helicobacter pylori* infection in China from 2014-2023: A systematic review and meta-analysis. *World J Gastroenterol.* (2024) 30:4636–56. 10.3748/wjg.v30.i43.4636 39575409 PMC11572641

[3] Sousa C Ferreira R Santos S Azevedo N Melo L. Advances on diagnosis of *Helicobacter pylori* infections. *Crit Rev Microbiol.* (2023) 49:671–92. 10.1080/1040841X.2022.2125287 36264672

[4] International Agency for Research on Cancer [IARC]. *Schistosomes, liver flukes and Helicobacter pylori*. IARC monographs on the evaluation of carcinogenic risks to humans. (Vol. 61). Lyon: IARC (1994). p. 1–241. PMC7681621 7715068

[5] Mentis A Boziki M Grigoriadis N Papavassiliou A. *Helicobacter pylori* infection and gastric cancer biology: Tempering a double-edged sword. *Cell Mol Life Sci.* (2019) 76:2477–86. 10.1007/s00018-019-03044-1 30783683 PMC11105440

[6] Clyne M Ó Cróinín T. Pathogenicity and virulence of *Helicobacter pylori*: A paradigm of chronic infection. *Virulence.* (2025) 16:2438735. 10.1080/21505594.2024.2438735 39725863 PMC12915409

[7] Muzaheed. *Helicobacter pylori* oncogenicity: Mechanism, prevention, and risk factors. *ScientificWorldJournal.* (2020) 2020:3018326. 10.1155/2020/3018326 32765194 PMC7374235

[8] Bakhti S Latifi-Navid S Safaralizadeh R. *Helicobacter pylori*-related risk predictors of gastric cancer: The latest models, challenges, and future prospects. *Cancer Med.* (2020) 9:4808–22. 10.1002/cam4.3068 32363738 PMC7333836

[9] Duan Y Xu Y Dou Y Xu D. *Helicobacter pylori* and gastric cancer: Mechanisms and new perspectives. *J Hematol Oncol.* (2025) 18:10. 10.1186/s13045-024-01654-2 39849657 PMC11756206

[10] Correa P Fontham E Bravo J Bravo L Ruiz B Zarama G Chemoprevention of gastric dysplasia: Randomized trial of antioxidant supplements and anti-*Helicobacter pylori* therapy. *J Natl Cancer Inst.* (2000) 92:1881–8. 10.1093/jnci/92.23.1881 11106679

[11] Huang R Choi A Truong C Yeh M Hwang J. Diagnosis and management of gastric intestinal metaplasia: Current status and future directions. *Gut Liver.* (2019) 13:596–603. 10.5009/gnl19181 31394893 PMC6860040

[12] Park S Kim Y Oh B Kang J. Risk factors for metabolic syndrome in the premetabolic state assessed using hierarchical clustering study in a health screening group. *Sci Rep.* (2024) 14:31169. 10.1038/s41598-024-82513-5 39732771 PMC11682037

[13] Liu M Liu S Lu Z Chen H Xu Y Gong X Machine learning-based prediction of *Helicobacter pylori* infection study in adults. *Med Sci Monit.* (2024) 30:e943666. 10.12659/MSM.943666 38850016 PMC11168235

[14] Wang B Xie R Qi W Yao J Shi Y Lou X Advancing Alzheimer’s disease risk prediction: Development and validation of a machine learning-based preclinical screening model in a cross-sectional study. *BMJ Open.* (2025) 15:e092293. 10.1136/bmjopen-2024-092293 39922598 PMC12107635

[15] Mochurad L Babii V Boliubash Y Mochurad Y. Improving stroke risk prediction by integrating XGBoost, optimized principal component analysis, and explainable artificial intelligence. *BMC Med Inform Decis Mak.* (2025) 25:63. 10.1186/s12911-025-02894-z 39920691 PMC11806876

[16] Tran V Saad T Tesfaye M Walelign S Wordofa M Abera D *Helicobacter pylori* (*H. pylori*) risk factor analysis and prevalence prediction: A machine learning-based approach. *BMC Infect Dis.* (2022) 22:655. 10.1186/s12879-022-07625-7 35902812 PMC9330977

[17] Zhu S Tan X Huang H Zhou Y Liu Y. Data-driven rapid detection of *Helicobacter pylori* infection through machine learning with limited laboratory parameters in Chinese primary clinics. *Heliyon.* (2024) 10:e35586. 10.1016/j.heliyon.2024.e35586 39170567 PMC11336724

[18] Smith S Boyle B Buckley M Costigan C Doyle M Farrell R The second Irish *Helicobacter pylori* Working Group consensus for the diagnosis and treatment of *Helicobacter pylori* infection in adult patients in Ireland. *Eur J Gastroenterol Hepatol.* (2024) 36:1000–9. 10.1097/MEG.0000000000002796 38829956 PMC11198963

[19] Sağlam NÖ Civan HA. Impact of chronic *Helicobacter pylori* infection on inflammatory markers and hematological parameters. *Eur Rev Med Pharmacol Sci.* (2023) 27:969–79. 10.26355/eurrev_202302_31190 36808372

[20] Mooney C Keenan J Munster D Wilson I Allardyce R Bagshaw P Neutrophil activation by *Helicobacter pylori*. *Gut.* (1991) 32:853–7. 10.1136/gut.32.8.853 1885065 PMC1378951

[21] Tohidpour A. CagA-mediated pathogenesis of *Helicobacter pylori*. *Microb Pathog.* (2016) 93:44–55. 10.1016/j.micpath.2016.01.005 26796299

[22] Karlsson A Ryberg A Dehnoei M Borch K Monstein H. Association between cagA and vacA genotypes and pathogenesis in a *Helicobacter pylori* infected population from South-eastern Sweden. *BMC Microbiol.* (2012) 12:129. 10.1186/1471-2180-12-129 22747681 PMC3520705

[23] Song Y Liu P Qi X Shi X Wang Y Guo D *Helicobacter pylori* infection delays neutrophil apoptosis and exacerbates inflammatory response. *Future Microbiol.* (2024) 19:1145–56. 10.1080/17460913.2024.2360798 39056165 PMC11529197

[24] Li C Yue J Ding Z Zhang Q Xu Y Wei Q Prevalence and predictors of *Helicobacter pylori* infection in asymptomatic individuals: A hospital-based cross-sectional study in Shenzhen, China. *Postgrad Med.* (2022) 134:686–92. 10.1080/00325481.2022.2085950 35653281

[25] Liu H Qin Y Yang J Huang G Wei X Wang L *Helicobacter pylori* infection as a risk factor for abnormal serum protein levels in general population of China. *J Inflamm Res.* (2022) 15:2009–17. 10.2147/JIR.S355446 35370414 PMC8968220

[26] Wu Y Zeng H Zhang M Li C Tang Y Li X Sex-specific risk factors associated with *Helicobacter pylori* infection among individuals undergoing health examinations in China. *Int J Gen Med.* (2022) 15:5861–8. 10.2147/IJGM.S367142 35791315 PMC9250778

[27] Jiao R Ma X Guo X Zhu Y Wu X Wang H Association of *Helicobacter pylori* infection and white blood cell count: A cross-sectional study. *BMJ Open.* (2024) 14:e080980. 10.1136/bmjopen-2023-080980 39488427 PMC11535675

[28] Li Y Choi H Leung K Jiang F Graham D Leung W. Global prevalence of *Helicobacter pylori* infection between 1980 and 2022: A systematic review and meta-analysis. *Lancet Gastroenterol Hepatol.* (2023) 8:553–64. 10.1016/S2468-125300070-5 37086739

[29] Wroblewski L Peek R Wilson K. *Helicobacter pylori* and gastric cancer: Factors that modulate disease risk. *Clin Microbiol Rev.* (2010) 23:713–39. 10.1128/CMR.00011-10 20930071 PMC2952980

[30] Zhang L Zhang D Wei L Zhou Y Li X Chen R *H. pylori* infection and osteoporosis: A large-scale observational and Mendelian randomization study. *BMC Infect Dis.* (2024) 24:305. 10.1186/s12879-024-09196-1 38475712 PMC10935925

[31] Liu J Wang Y Zhao Q Luo R Xiao M Zhang M Prevalence and risk factors for *Helicobacter pylori* infection in southwest China: A study of health examination participants based on 13C-urea breath test. *Turk J Med Sci.* (2017) 47:1456–62. 10.3906/sag-1605-149 29151317

[32] Liang C Zhou C Pan J. Dietary patterns and *Helicobacter pylori* infection: Insights and future research. *Asian J Surg.* (2024) 6:32334. 10.1016/j.asjsur.2024.11.104 39613639

[33] Malfertheiner P Megraud F Rokkas T Gisbert J Liou J Schulz C Management of *Helicobacter pylori* infection: The Maastricht VI/Florence consensus report. *Gut.* (2022) 71:1724–62. 10.1136/gutjnl-2022-327745 35944925

[34] Wang L Li Z Lai J Si Y Chen J Chua E Risk factors associated with *Helicobacter pylori* infection in the urban population of China: A nationwide, multi-center, cross-sectional study. *Int J Infect Dis.* (2025) 154:107890. 10.1016/j.ijid.2025.107890 40096882

